# Imaging findings of vertebral osteomyelitis caused by nontuberculous mycobacterial organisms: Three case reports and literature review

**DOI:** 10.1097/MD.0000000000029395

**Published:** 2022-06-17

**Authors:** Xiao Jing Yu, Yu Dong Lin, Peng Hu, Chi Shing Zee, Shu Juan Ji, Fei Zhou

**Affiliations:** a Department of Radiology, Sir Run Run Shaw Hospital, School of Medicine, Zhejiang University, Hangzhou, Zhejiang, China; b Department of Urology, Sir Run Run Shaw Hospital, School of Medicine, Zhejiang University, Hangzhou, Zhejiang, China; c Department of Radiology, USC University Hospital, Los Angeles, CA; d Department of Infectious Disease, Sir Run Run Shaw Hospital, School of Medicine, Zhejiang University, Hangzhou, Zhejiang, China.

**Keywords:** atypical mycobacteria, nontuberculous mycobacteria, vertebral osteomyelitis

## Abstract

**Rationale:**

Prompt diagnosis of nontuberculous Mycobacterial (NTM) vertebral osteomyelitis is challenging, yet necessary to prevent serious morbidity and mortality. Here, we report 3 cases of vertebral osteomyelitis caused by NTM with imaging findings.

**Patient concerns:**

Case 1, a 58-year-old male patient, was admitted to our hospital because of the presence of a pulmonary mass for 6 months with cough and chest pain.

Case 2, a 50-year-old male patient, had fever and cough for 3 years and was diagnosed with tuberculosis. Antituberculosis treatment was ineffective, accompanied by lymph node enlargement and osteosclerotic changes involving vertebral bodies.

Case 3, a 66-year-old female patient, was admitted to our hospital with a mass on the top of her head for 1 month, which ruptured in the last 2 weeks.

**Diagnoses:**

Case 1: Sputum culture revealed *Mycobacterium* (*M.*) *avium.*

Case 2: The final culture results of the lymph node biopsy samples were *M. intracellulare.*

Case 3: Culture results of the sputum and pus from the abscess were *M. gordon*.

We found sclerosing lesions in the spine in all 3 NTM patients, which were easily misdiagnosed as metastatic tumors. In 2 cases, there was bone destruction in the ilium with limbic sclerosis, and there were abscesses near the ilium and in front of the sacrum in 1 case.

**Interventions:**

Case 1 was transferred to other specialist hospital.

Case 3 received surgical treatment for cranial lesions and abscess drainage.

Case 2 and case 3 received targeted treatment for nontuberculous mycobacteria in our hospital.

**Outcome:**

The condition of case 1 was unknown.

Recovery of case 2 was uneventful because of prolonged illness; however, inflammation gradually improved overall.

Case 3 had no recurrence following surgical treatment.

**Lessons:**

In our 3 cases of NTM vertebral osteomyelitis, bone lesions were often misdiagnosed as bony metastases because of the presence of multiple sclerotic lesions. Diagnoses were challenging and delayed. It is important to consider osteomyelitis by NTM when disseminated osteosclerosis with or without osteolytic bone lesions is present in conjunction with continuous inflammatory symptoms and signs. Moreover, an open biopsy of the lesion should be performed for a definitive diagnosis.

## 1 Introduction

Nontuberculous Mycobacterium (NTM) refers to all *Mycobacterium*, except *Mycobacterium* (*M.*) *tuberculosis* (TB) and *M. leprae*, and is also known as environmental mycobacterium. NTM is readily isolated from environmental sources, such as water, dust, and soil, and infections are presumed to occur via the respiratory or gastrointestinal tract.^[1,2]^ In general, this mycobacterium is less pathogenic to humans than is Mycobacterium TB; however, if there is a susceptibility factor, local or systemic immune dysfunction of the host can lead to infectious lesions.^[1–3]^ In addition, human-to-human transmission is rare.^[4]^

NTM can invade human lungs, bones, joints, lymph nodes, skin, soft tissue, and other tissues and cause systemic disseminated diseases. In many low-burden TB countries, the incidence of NTM has surpassed that of TB.^[5]^

Clinically, the diagnosis and differential diagnosis of spinal NTM disease are challenging because of the currently limited understanding of the condition. Improving our understanding of the imaging features of spinal NTM infection is valuable for accurate diagnoses and timely treatment of NTM. According to our experience and the literature,^[6]^ the diagnosis of NTM osteomyelitis is often delayed and misdiagnosed, which results in delayed treatment. Imaging reports of vertebral osteomyelitis with NTM are scarce. Here, we report imaging findings of 3 cases of vertebral osteomyelitis with NTM. We have summarized our imaging findings of NTM vertebral osteomyelitis, alongside typical clinical histories to facilitate prompt and accurate diagnoses in the future.

## 2 Case presentation

### 2.1 Case 1

The patient was a 58-year-old man who was admitted to our hospital because of the presence of a pulmonary mass for 6 months and a clinical history of cough and chest pain for more than 10 days. The patient had a fever of 38°C during hospitalization. The patient had no specific underlying disease, had never undergone surgery, and had an unremarkable family history. Laboratory findings showed that white blood cell count was 14.2 × 10^9^/L, the percentage of neutrophile granulocyte was 82.4%, red blood cell count was 3.74 × 10^12^/L, hemoglobin level was 92 g/L, C-reactive-protein level was 156.4 mg/L, and erythrocyte sedimentation rate (ESR) was 108 mm/h. Serum carbohydrate antigen 199, carbohydrate antigen 125, alpha- fetoprotein, carcinoembryonic antigen, and prostate-specific antigen were negative.

**Figure 1:**
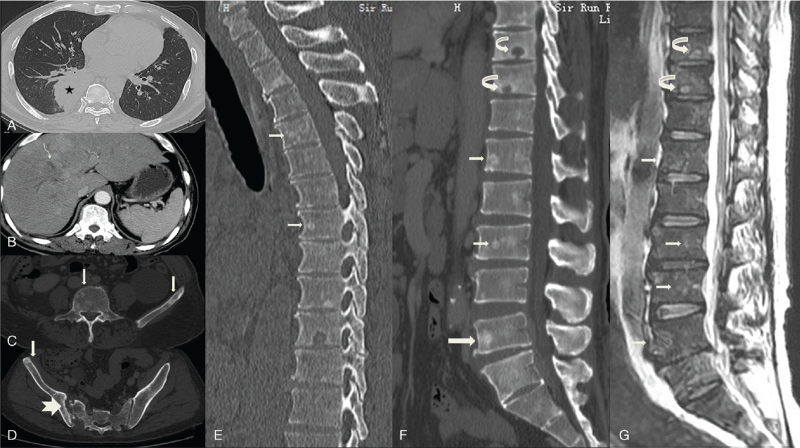
(A) Soft tissue mass in the right lower lung (black star). (B) A low-density mass (thin curved arrow) can be seen near the hepatic hilum, accompanied by distant bile duct dilatation. (C-F) Multiple sclerotic foci can be seen in the thoracolumbar spine and ilia: some small circular high-density lesions (thin straight arrow), partly agglomerated high-density lesions with an ill-defined margin (thick straight arrow), and partial round destruction of the endplates with a sclerotic margin (curved arrow). (D) Note the destruction of bone on the right sacroiliac articular surface, with irregular and sclerotic margins (swallow tail arrow). (G) Sagittal T2-weighted imaging shows high signal intensity of the lesion (thin straight arrow), high signal intensity of the rounded lesions at the endplate with low signal margins (curved arrow), and no signal reduction or other degenerative manifestations in the adjacent intervertebral disk.

The patient underwent computed tomography (CT) scans of the chest and whole abdomen. The chest CT scan (Fig. 1A) showed a right lower lung mass with a fuzzy edge, which was considered a tumor, accompanied by inflammation, revealed by enlarged mediastinal lymph nodes. The contrast-enhanced CT scan of the abdomen (Fig. 1B) demonstrated a low-density shadow near the porta hepatis, accompanied by distant bile duct dilation. An magnetic resonance imaging (MRI) with enhancement and Magnetic Resonance Cholangiopancreatography of the liver showed a hilar mass with intrahepatic bile duct dilation; thus, bile duct cell carcinoma was suspected.

On the CT scans of the chest and whole abdomen, abnormalities of the vertebral bodies were simultaneously observed. Multiple sclerotic lesions were found in the thoracic and lumbar vertebrae and the iliac bone with small circular high-density lesions and clumpy high-density shadows with blurred edges; moreover, rounded bone destruction could be seen on the lower edge of the T10 and T11 vertebral bodies, accompanied by a sclerotic margin that mimicked Schmorl node (Fig. 1C-F). In addition, in the right sacroiliac joint, we observed osteolytic bone destruction on the iliac surface with irregular sclerotic margins (Fig. 1D). Lumbar MRI showed a general reduction in the signal of vertebral bodies, and a reversal of red bone marrow was considered (red blood cell-related indices were all reduced). Furthermore, abnormal intensities with slightly higher signal on T2-weighted imaging (T2WI) in multiple vertebral bodies and accessories were also detected (Fig. 1G), and there was no intervertebral disc involvement.

The patient was suspected of malignancy due to the presence of a hilar mass and multiple sclerosing lesions in the spine on imaging. Therefore, the patient underwent bone scans and several needle biopsies. Bone scintigraphy showed intense uptake of multiple cervical, thoracic, and lumbosacral vertebrae, the bilateral clavicle, the humerus and ribs, bilateral shoulder joints and scapulae, and bilateral sacroiliac joints and femurs; thus, multiple bone metastases were considered. Both bronchoscopy and lung biopsy showed chronic active inflammation. Iliac biopsy showed proliferation of fibrous tissue in the bone marrow cavity, accompanied by a large number of chronic inflammatory cells and a small number of neutrophils. Liver biopsy showed small pieces of liver tissue with focal necrosis and inflammatory cell infiltration. Fibrotic hyperplasia with acute and chronic inflammatory cell infiltration was also observed.

The patient reported finding skin nodules at the root of the right thigh and below the groin. Skin biopsy was positive for acid-fast staining. The patient was transferred to the TB control specialist hospital. The patient's sputum culture revealed *M. avium*.

### 2.2 Case 2

A 50-year-old male patient was admitted to hospital because of recurrent fever for 3 years and chest pain for more than 1 year. The patient began experiencing fever and cough 3 years ago and was diagnosed with TB according to a positive acid-fast lymph node biopsy at another hospital. Anti-TB treatment was administered; however, their fever continued and was accompanied by lymph node enlargement and osteosclerotic changes involving the vertebral bodies. Positron emission computed tomography showed enlargement of multiple lymph nodes and increased fluorodeoxyglucose metabolism in multiple bones in the body, which was considered malignant. The patient visited other hospitals numerous times, and multiple biopsies (bone, lung, and bronchial biopsies) were performed because of suspected malignancy. However, no evidence of tumors or other diseases was found.

Laboratory finding showed that white blood cell count was 16.4 × 10^9^/L, the percentage of neutrophile granulocyte was 83.5%, hemoglobin level was 94 g/L, C-reactive-protein level was 147.32 mg/L, procalcitonin was 0.33 ng/mL, and ESR was 51 mm/h.

**Figure 2:**
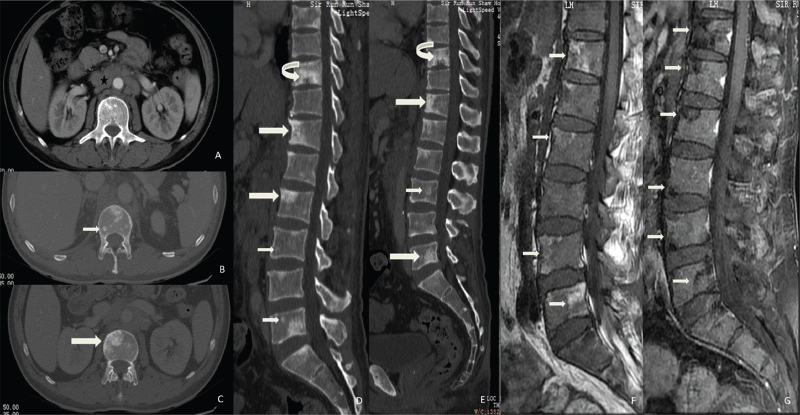
(A) Enlargement of multiple retroperitoneal lymph nodes can be seen (black star). (B-E) Multiple sclerotic lesions can be seen in the vertebral bodies: some are small ring-like high-density lesions (thin straight arrow), some are agglomerated high-density lesions with an ill-defined margin (thick straight arrow), and some appear to erode endplates (curved arrow). (F) After 5.5 months of treatment, T1-weighted imaging showed lesions gradually changing into yellow marrow, which suggested improvement (thin straight arrow). (G) After continued treatment for a further 3 months, fat saturation T1-weighted imaging with contrast showed that the lesions with yellow marrow were suppressed and did not enhance, which indicated recovery (thin straight arrow).

The enhanced abdominal CT (Fig. 2A) showed multiple enlarged lymph nodes in the retroperitoneum. In the thoracolumbar vertebrae, multiple sclerotic lesions were observed (Fig. 2B-E), with ill-defined margins and multiple endplate involvement. Several small circular sclerotic foci, similar to those of case 1, were also detected.

MR of the left ankle showed multiple nodules or patchy hyperintensity on T2WI fat-saturation images of the left ankle and various bones of the left foot.

Retroperitoneal lymph node biopsy showed positive acid-fast staining, and the final culture results of lymph node biopsy samples were *M. intracellulare*.

After 5.5 months of treatment, the T1-weighted MR examination showed that the vertebral lesions had gradually changed to yellow bone marrow (Fig. 2F), and the lesions did not show any enhancement (Fig. 2G) after an additional 3 months of treatment, which indicated that the treatment was effective.

### 2.3 Case 3

**Figure 3:**
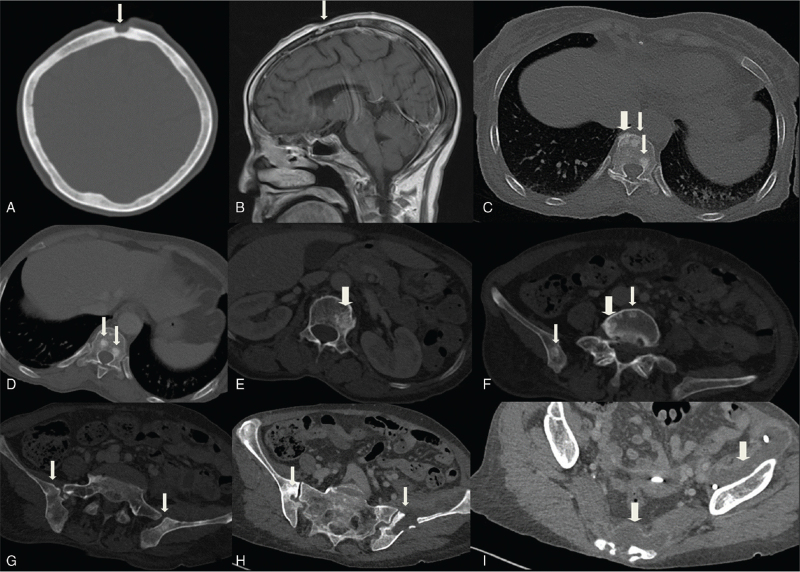
(A) Soft tissue defect of the forehead with local destruction of the frontal bone (arrow). (B) Sagittal T1-weighted imaging with enhancement shows plaque enhancement of the destruction of the skull (arrow). (C-G) Multiple sclerotic lesions were found in the vertebral bodies and both sides of the iliac bones that presented as nodules or clumps of high-density shadows (straight arrows), and lesions at the edge of the vertebral body showed cortical sclerosis and irregularity (thick straight arrows). (F) Internal low-density shadows can be seen in part of the lesion (thin straight arrows). (H-I) Both sacroiliac joints showed bony destruction and abscess formation in front of the left iliac bone and sacrum (thick straight arrows; a drainage tube can be seen inside the abscess).

The patient was a 66-year-old woman who was admitted to our hospital with a mass on the top of her head for 1 month that ruptured in the last 2 weeks and a fever of 39°C for 4 days. The patient had a history of chronic hepatitis B and cholecystolithiasis. Laboratory findings showed that white blood cell count was 11.4 × 10^9^/L, red blood cell count was 2.69 × 10^12^/L, the percentage of neutrophile granulocyte was 75.4%, hemoglobin level was 70 g/L, C-reactive-protein level was 112.7 mg/L, procalcitonin was 0.24 ng/mL, and ESR was 101 mm/h. On physical examination, a scalp defect of approximately 20 × 20 mm involving the skull was observed at the top of the patient's head, and purulent exudation was observed in the cavity. Skull CT and MRI (Fig. 3A and B) showed local scalp defect of the forehead, with swelling of the surrounding soft tissue and destruction of the adjacent bone. MRI with contrast showed residual soft tissue enhancement, and nodular enhancement was observed at the site of bone destruction. Surgery was performed on the lesion of the skull, and postoperative pathology revealed granulomatous inflammation. After surgery, the patient noticed a supple mass in the left groin and developed a fever.

Whole abdominal enhanced CT (Fig. 3C-G) revealed multiple hyperdense lesions on thoracolumbar vertebral bodies with ill-defined margins. Several small circular sclerotic foci, similar to those seen in cases 1 and 2, were also detected. There was destruction of the surface of the iliac bone on both sides of the sacroiliac joints (Fig. 3H) with a clear boundary. Abscess formation was observed in front of the left ilium and sacrum (Fig. 3I). Lumbar MR showed slightly high signal on T2WI, moderate enhancement after enhancement, and no intervertebral disc involvement.

Drainage of the abscess was performed. The sputum was positive for acid-fast staining. Culture results of the sputum and pus from the abscess in front of the ilium revealed *M. Gordon*.

## 3 Discussion

The most common clinical manifestation of NTM infection is pulmonary disease with rare extrapulmonary manifestations, such as lymphadenitis and soft tissue infection. NTM vertebral osteomyelitis is exceedingly rare.

It is generally acknowledged that people with immunodeficiency or long-term hormone use are susceptible to NTM infections. However, in our cases, except hepatitis B in case 3, there were no underlying diseases. Garcia et al^[7]^ reviewed a total of 28 publications that comprised 41 cases of vertebral osteomyelitis caused by NTM and found that most patients did not present with a significant underlying condition (31%) or causative factor (36.5%). In the review by Shinohara et al,^[8]^ about half of all NTM spondylitis cases occurred in healthy people. Blunt-force trauma was a risk factor for NTM spondylitis, and it was hypothesized that macrophages, which phagocytose NTM, migrate to the injury site for tissue restoration but release the pathogen, resulting in the development of vertebral osteomyelitis. Moral et al^[9]^ also reported a case of NTM spondylitis with normal immunity. Gray et al^[10]^ reported a case who underwent an extended period of corticosteroid therapy and proposed that chronic corticosteroid therapy poses a greater risk for the development of NTM vertebral osteomyelitis. These findings demonstrate that a diagnosis of NTM spondylitis should not be ignored because of a patient's normal immunity.

Garcia et al^[7]^ reported that the mean age of patients with vertebral osteomyelitis caused by NTM was 46.2 ± 18 years, and prevalence was higher in men (53.6%). Our 3 cases of NTM osteomyelitis of 2 males and 1 female aged 50 to 66 years are consistent with the review by Garcia et al.

According to the report by Garcia et al,^[7]^ the most commonly reported agent in NTM vertebral osteomyelitis was *M. avium*, followed by *M. xenopi*. Kim et al^[11]^ reported 6 cases and reviewed 63 cases of vertebral osteomyelitis caused by NTM and found that the most common species, regardless of the presence of HIV co-infection, was the *M. avium* complex, followed by *M. xenopi*. The Runyon classification divides NTM into 4 groups according to colony pigment and growth rate: photochromogen, scotochromogen, non-photochromogen, and rapid grower. *M. avium* or *M. avium*-intracellulare complex (MAC), together with *M. intracellulare*, are classified into Runyon group III non-photochromogenic *Mycobacterium*. MAC, contains 2 types of Mycobacteria: *M. avium* and *M. intracellulare*.^[1]^ Although molecular techniques can identify MAC isolates as *M. avium* or *M. intracellulare*, they provide no prognostic or therapeutic advantage. In our report, 1 case had an *M. avium* infection, another had an *M. intracellulare* infection, and the third case had an *M. Gordon* infection. The source of infection in our report is consistent with the abovementioned studies.

Table 1 summarized the clinical and imaging details of cases 1 to 3. The 3 cases of spinal NTM infection demonstrated clear radiographic features. The lesions of NTM infection in the spine were all sclerosed and disseminated in multiple vertebrae. Furthermore, the lesions showed the following pattern of increased bone density: small circular sclerotic foci and clumpy high-density shadows with blurred edges. Yamashita et al^[12]^ reported a case of multiple osteomyelitides due to *M. avium* infection, and CT revealed osteosclerotic lesions in the left femur and lumbar vertebrae, which was initially suspected by the radiologist as multiple malignant bone metastases. Liang et al^[13]^ summarized the CT findings of 21 patients with NTM spondylitis and reported the following CT features: involvement of more than 3 segments of the vertebral body, osteogenic bone destruction, and multi-segment polymorphic bone destruction without intervertebral disc involvement. This is largely consistent with the imaging findings of our cases. NTM causes osteogenic bone destruction, which may be related to bone repair; NTM causes nonspecific chronic inflammation and chronic stimulation of the bone, which leads to reactive osteosclerosis, and sclerosis prevents the vertebra from collapsing. The toxicity of NTM is low, the lesions caused by NTM are confined to the vertebral body, and the intervertebral disc is surrounded by the annulus fibrosus; therefore, it is difficult for NTM to break through the annulus fibrosus and affect the intervertebral disc.

**Table 1:** Summary of clinical details of cases 1 to 3.

	Case 1	Case 2	Case 3
Sex	Male	Male	Female
Age	58	50	66
Underlying disease	None	None	Chronic hepatitis B, cholecystolithia
Time from onset to diagnosis	8 mo	3 yrs	Almost 2 mo
Fever	Yes	Yes	Yes
Results of laboratory tests			
White blood cell count	Increase	Increase	Increase
Percentage of neutrophile granulocyte	Increase	Increase	Increase
Red blood cell count	Decrease	Decrease	Decrease
Hemoglobin level	Decrease	Decrease	Decrease
C-reactive-protein level	Increase	Increase	Increase
Erythrocyte sedimentation rate	Increase	Increase	Increase
Vertebra			
Distribution	Multiple, disseminated	Multiple, disseminated	Multiple, disseminated
Sclerosing lesion			
Small circular high-density lesions	Yes	Yes	Yes
Clumpy high-density shadows with blurred edges	Yes	Yes	Yes
Endplate involved	Yes	Yes	None
Intervertebral disk involved	None	None	None
Sacroiliac joint	Right sacroiliac joint, osteolytic bone destruction on the iliac surface with irregular sclerotic margins	Normal	Both sacroiliac joints, destruction of the surface of iliac bone, with clear boundary
Abscess	None	None	Abscess formation in front of the left ilium and sacrum
Bone scintigraphy or PET-CT	Bone scintigraphy showed intense uptake in multiple vertebrae and other bones	PET-CT showed enlargement of multiple lymph nodes, increased FDG metabolism, and increased FDG metabolism in multiple bones	Not performed
Other imaging findings	A right lower lung mass; a mass near the porta hepatis with intrahepatic bile duct dilation	Retro-peritoneal lymphadenopathy	Local scalp defect of the forehead, with swelling of the surrounding soft tissue and destruction of the adjacent bone
Skin	Skin nodules at the root of the right thigh and below the groin	Normal	A mass on the top of head for 1 mo, and ruptured in the last 2 wk
Diagnosis	Skin biopsy tissue was positive for acid-fast staining, sputum culture revealed *M. avium*	Retroperitoneal lymph node biopsy showed positive acid-fast staining, and the final culture results of lymph node biopsy samples were *M. intracellulare*	Sputum was positive for acid-fast staining, culture results of sputum and pus from the abscess in front of the ilium were *M. gordon*

FDG = fluorodeoxyglucose, *M. = Mycobacterium*, PET-CT = positron emission computed tomography.

Liang et al^[13]^ did not specifically mention the low-density shadow in nodular sclerosis; however, it could be seen in the images provided in their report. We believe that small circular sclerotic foci are an early sign of infection, whereby the lesion initially appears as a small ring-like area of sclerosis. The gradual increase in lesion size leads to the small ring-like sclerotic lesion evolving into a larger cluster with a high density and an ill-defined margin. If the sclerotic lesion occurs in the anterior margin of the vertebral body, the anterior margin of the vertebral body appears highly irregular. With careful observation, the low-density dots could be seen inside most of the high-density lesions. Furthermore, these low-density speckles inside the sclerotic lesions did not have high signal on T2WI. This suggested that the necrotic material inside the large lesions were not pure water or pus; rather, they may have been the caseous material of TB.

If the lesion occurred in the vertebral endplate or articular surface, we observed bone destruction, accompanied by a sclerotic edge, which suggested an indolent destruction process. In the case of vertebral endplate infection, initially, the edge of the vertebral body became irregular, followed by bone destruction of the irregular margin, which should be distinguished from vertebral endplate osteochondritis and Schmorl node. Vertebral endplate osteochondritis and Schmorl node started in the degenerative disc, and the disc signal was reduced and protruded into the adjacent endplate. However, we observed normal MRI signal in the disc in the NTM osteomyelitis. In addition, MRI showed that the signal of the diseased bone on T2WI was not excessive, which may be related to the sclerosis, and the enhancement was mild.

In the review by Daphne et al,^[6]^ osteomyelitis caused by NTM was found to resemble acute pyogenic infection but with slower progression. That is, NTM vertebral infection could result in bone destruction of the articular surface, accompanied by marginal sclerosis, and may be associated with abscess formation. This is consistent with the more severe manifestations in our cases (involvement of the vertebral endplate in cases 1 and 2 and the sacroiliac joint in cases 1 and 3). Moreover, Daphne et al found that additional features of spine infection may include the involvement of only a single end plate and noncontiguous levels. Indeed, we found the same patterns in cases 1 and 2. Liang et al^[13]^ compared the radiographic findings of NTM spondylitis with those of spinal TB. Because of the strong virulence of Mycobacterium TB, spinal TB is more likely to involve the 2 adjacent vertebrae and damage adjacent structures, such as intervertebral discs or paravertebral soft tissues; moreover, the paravertebral abscesses are likely to be more extensive. In contrast, NTM spondylitis rarely shows these symptoms.

In our cases, in addition to the vertebral and sacroiliac joints, other bones were also involved, such as the ilium. However, the lesions were predominantly sclerotic.

Although the number of cases in our study was small, the imaging findings of the 3 cases had distinct features of NTM vertebral osteomyelitis that have not been described previously. Firstly, when the lesions were small, they presented as small annular or nodular sclerosis. Secondly, larger lesions presented as large sclerotic masses with ill-defined margins. Further progression of the lesions may result in destruction of the vertebral endplates or articular bone (especially in the sacroiliac joint), and in more severe cases, abscess formation could be observed in the adjacent soft tissue. Such progression of infection could be observed simultaneously in 1 patient in whom the vertebral body lesions were multiple and disseminated, and the discs were not involved.

In our 3 cases of NTM vertebral osteomyelitis, bone lesions were often misdiagnosed as bony metastases owing to the presence of multiple sclerotic lesions. The diagnoses were challenging and delayed, with the longest delay in diagnosis of 3 years in case 2. In addition, cases 1 and 2 were required to undergo excessive invasive tests in order to obtain an accurate diagnosis.

Thus, accurate and prompt diagnosis and effective treatment of NTM infection of the vertebrae are crucial to prevent severe bone destruction and neurological complications. In conclusion, NTM vertebral infection should be considered when multiple lesions (small or large) with osteosclerosis and end plate destruction (or articular surface destruction) with sclerotic edges are observed alongside persistent inflammatory symptoms (e.g., pulmonary inflammation, skin nodules, retroperitoneal lymph node enlargement, increased markers of inflammation in the laboratory, and fever).

## Author contributions

**Clinical data curation:** Shu Juan Ji.

**Data curation:** Peng Hu, Shu Juan Ji, Xiao Jing Yu, Yu Dong Lin.

**Imaging data curation:** Xiao Jing Yu, Yu Dong Lin, Peng Hu.

**Methodology:** Fei Zhou, Peng Hu.

**Writing – original draft:** Xiao Jing Yu, Yu Dong Lin.

**Writing – review & editing:** Chi Shing Zee.
